# First Step to Equity and Organization in Waiting Lines for Stone Surgery. Translation of Prioritization Scores

**DOI:** 10.1590/S1677-5538.IBJU.2025.0039

**Published:** 2025-03-29

**Authors:** Guilherme Gentile, Fabio Carvalho Vicentini, Erik Montagna, Daniel Pérez-Fentes, Guilherme Pimenta Roncete, Artur Henrique Brito, Fabio Miranda Torricelli, Alexandre Danilovic, Carlos Alfredo Batagello, Eduardo Mazzucchi, William Carlos Nahas

**Affiliations:** 1 Faculdade de Medicina da Universidade de São Paulo Divisão de Urologia São Paulo SP Brasil Divisão de Urologia, Faculdade de Medicina da Universidade de São Paulo - FMUSP, São Paulo, SP, Brasil; 2 Hospital Municipal Brigadeiro Serviço de Urologia São Paulo SP Brasil Serviço de Urologia, Hospital Municipal Brigadeiro, São Paulo, SP, Brasil; 3 Centro Universitário da Faculdade de Medicina ABC - FMABC Pós-Graduação Pesquisa e Inovação Santo André Brasil Pós-Graduação, Pesquisa e Inovação, Centro Universitário da Faculdade de Medicina ABC - FMABC, Santo André, Brasil; 4 Complexo Hospitalar Universitário de Santiago de Compostela Espanha Complexo Hospitalar Universitário de Santiago de Compostela, Espanha; 5 Faculdade de Medicina da Universidade de São Paulo São Paulo SP Brasil Faculdade de Medicina da Universidade de São Paulo – FMUSP, São Paulo, SP, Brasil

**Keywords:** Kidney Calculi, Urolithiasis, Nephrolithotomy, Percutaneous

## Abstract

**Purpose:**

The use of patient prioritization tools is one of several methods to enhance the management of waiting times for elective surgeries. Developing these tools specifically for urinary stones in the Brazilian context may enhance queue management and increase patient satisfaction. This study aims to adapt two previously published scores, the WCWL (Western Canada Waiting List - general criteria) and the SCQ-score (specific to urinary stone criteria), into Brazilian Portuguese.

**Materials and Methods:**

Our study adhered to established protocols for the cross-cultural adaptation and translation of health-related questionnaires. The process for translating both original scores involved four steps: initial translation, back-translation, committee review, and pre-testing. The translations were conducted by professionals proficient in the relevant languages. The pre-test phase engaged eight endourologists who applied the translated versions of the scores to twelve hypothetical patient cases.

**Results:**

Our study successfully produced Brazilian Portuguese versions of the SCQ and WCWL scores. During the pre-testing, these scores were found to be quick to perform (with an average completion time of 1 minute and 35 seconds) and were deemed easy to understand and use by the endourologists. However, there was a concern regarding the practical utility and interpretability of the WCWL score due to its more generalized criteria.

**Conclusion:**

We successfully developed the Brazilian Portuguese version of the Western Canada Waiting List and SCQ-score. This development will allow further studies to evaluate the impact of their use within the Brazilian healthcare environment.

## INTRODUCTION

Long waiting lists for surgical procedures are a serious problem that occurs when the demand for surgery exceeds the resources available in health centers. This is especially common in countries with publicly funded health systems, like Canada, Spain, New Zealand, and the United Kingdom (1). In Brazil, the Federal Medicine Council estimated that over 900,000 Brazilians were waiting for surgery, with a waiting time of up to 10 years (2). This delay in treatment leads to a whole new set of problems, with significant consequences in economics, ethics, and health terms such as development of complications that could have been prevented with timely procedures (3-5), dissatisfaction of patients, increased pain and decreased quality of life (6, 7) . Among the diseases that require surgery and have long waiting times, kidney stones deserve special attention. Kidney stones have a high prevalence in Brazil, often involve expensive procedures and materials for surgery and, if left untreated, can lead to severe complications such as renal failure, recurrent infections, and chronic pain (8, 9). In our institution alone, nearly 400 patients wait for urinary stones surgery, with waiting times that can exceed a year.

Since gathering enough resources to supply this high demand for procedures is a tough – and sometimes impossible - task, adequate management of waiting lines is crucial. To this day there is no absolute agreement on how these lines should be organized. In Brazil, usually patients are listed in a "first come, first serve" manner, in which patients are organized according to their time on the waiting list, and sometimes they are classified in groups of priority: methods that do not fully address all the issues in waiting lines. In this setting, implementing effective prioritization criteria can benefit both patients and healthcare centers involved in managing long waiting lines, providing fair, equitable and transparent criteria (10). However, developing reliable and valid prioritization scores is a complex process that requires research, a thorough discussion/debate among experts, validation, and translation efforts. Therefore, translating already existing prioritization scores is a justified approach to address the urgent need for better management of patients waiting for elective surgery for urinary stones in Brazil.

To develop prioritization criteria, Taylor et al. (11) developed a score composed of five questions that addressed pain, quality of life impairment and presence of complications related to the disease in order to determine prioritization. The final score in these questions would range from 0 to 55 (with higher scores being the most critical patients) and it was developed to organize all patients that needed treatment in an operating room, irrespective of the disease. This score was developed along with other disease-specific scores, in a federally funded partnership of organizations in Canada, named Western Canada Waiting List Project (WCWL). More recently, Pérez et al. (12) developed a new prioritization score specifically for urinary stones, called SCQ-Score. This tool is composed of nine questions regarding symptoms, presence of complications and urinary stents, kidney function and stone characteristics. The final score ranges from 0 to 18, with higher scores indicating more prioritized patients.

Our study aims to accomplish the translation and cross-cultural adaptation to Brazilian Portuguese of both WCWL and SCQ scores, as well as their application time, making it possible to evaluate their use in practice and foster future studies.

## MATERIALS AND METHODS

**Figure 1:**
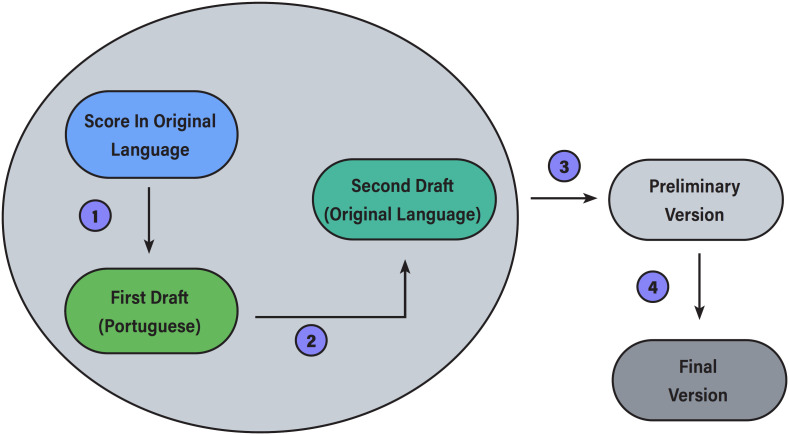
Flowchart of Translation Process.

Our study follows well-established recommendations (13) regarding methods for translation and cross-cultural adaptation of health-related questionnaires. These recommendations consist of four crucial steps that should be taken during the translation process (Figure-1).

1. **Initial Translation:** Two professionals translate the original score, independently. The comparison of their two versions generates the first draft.
2. **Back-translation:** the first draft is translated back into the original language by two other translators independently.
3. **Authors compare the original score and both drafts:** all drafts and the original version are compared, creating a preliminary version.
4. **Pre-test:** the preliminary version is submitted to a pre-test (12 fictional cases in our study). After the necessary corrections, the final version is generated.

First, the original score is translated into Brazilian Portuguese by two professionals (both authors of this study), independently, who have knowledge of the score's subject and speak English (WCWL score's original language) and Spanish (SCQ score's original language). Then, both translated versions are compared, and any discrepancies are solved through discussion, creating the first draft of the translation.

In the second step, this first draft is translated back into the score's original language, and then compared to the original score. This is done by two other independent translators who were not involved in the study, don't have knowledge of the scores’ purpose, speak Brazilian Portuguese fluently and have the same native language in which the original score was published. The purpose of this step, called back-translation, is to identify if meaning was lost or misunderstood in the initial translation.

Finally, all versions created in the first two steps are compared by the two authors / translators. If any discrepancies are found in this step, they are solved by discussion and consensus. If no agreement was reached, the matter is resolved with the aid of a third professional, also experienced in the subject of the scores and all languages involved. This process results in a preliminary version for each score (SCQ and WCWL), which is adapted to the Brazilian culture and maintain the characteristics of the original scores.

After these steps, the preliminary version of each score is pre-tested. In our study, we created a set of 12 fictional patient scenarios reflecting the cases on our waiting list. These fictional cases contained relevant information for the application of the scores and clinical decision making. Eight endourologists were selected to apply the translated scores to the fictional patients during a closed meeting. Sentences with ambiguous meanings, the time required to apply the scores, dubious questions and all other eventual difficulties that could prevent the daily use of the scores were noted and discussed. After this final step, we achieved the final versions of the scores: WCWL-BR and SCQ-BR (supplementary material).

## RESULTS

During the translation process, all points of discussion between different versions of the scores were discussed and settled by the two main authors / translators, without the need for a third-party evaluation. The pre-test was conducted in a closed group meeting in which all eight selected urologists, the main author and a facilitator were present. The participants were previously instructed about the purpose of the scores and how to use them. All fictional cases (presented in the supplementary material) were displayed in a slideshow, and all participants were asked to classify each patient based on the translated versions of WCWL and SCQ scores. The average time to classify each patient was 1 minute and 35 seconds for both SCQ and WCWL scores. No disagreement occurred between the participants while rating each criterion in the scores, but they agreed that WCWL's criteria were more prone to errors due to subjectivity. Also, no specialist objected to the values assigned to each criterion by the original score's authors. After the pre-test meeting, no alterations were needed in the preliminary versions.

## DISCUSSION

Prioritization scores for the management of patients waiting for elective surgery have seen some advancements in recent years, especially after the COVID-19 pandemic (14), which significantly hindered elective surgeries and added a large number of patients to already long waiting lines. The importance of establishing criteria for determining the priority of patients on waiting lists for surgery has been recognized more than ever, as they can help improve decision-making processes, ensure fairness and transparency, and facilitate resource allocation. However, there are several difficulties associated with applying prioritization scores in clinical practice.

One of the main challenges is determining which criteria are relevant and should be included in the scoring system. The development of new criteria requires extensive research and evidence gathering involving multidisciplinary teams, which is laborious and time-consuming (15). Moreover, it is difficult to address all possible variables that could impact a patient's urgency for surgery, leading to potential bias or oversimplification in the scoring process. Despite these challenges, prioritization scores have shown promise in streamlining the management of surgery waiting lists and providing a more objective approach to categorizing patients based on their clinical needs. Our study allows future research in this field that validate WCWL and SCQ scores within the Brazilian context. Also, by overcoming the language barrier, we also create opportunities for Brazilian researchers and healthcare professionals to use these tools and advance this field of knowledge in their own environment.

One of the strong points of our study was our rigorous adherence to a well-established methodology in the translation and cultural adaptation process, using adequate translators for each step of the process. By adhering to these methods, we can preserve the reliability and usability of the original scores in the Brazilian context, while making them accessible and applicable to clinical practice. Another strength of our study is that it provides, to our knowledge, the first prioritization tool for kidney stones in Brazilian Portuguese. This fills an important gap in the existing literature and clinical practice, as it allows healthcare providers in Brazil to better manage and prioritize patients waiting for surgery.

However, it is important to acknowledge the limitations of our study. First, we chose not to retest the internal validity of the translated scores. While this decision may raise questions about the reliability of the scores, we followed established translation and cultural adaptation guidelines, which are widely accepted as best practices. Additionally, there is debate in the literature over whether this step is essential, since there are authors who argue that the internal validation of these tools is preserved when translation processes are done accurately (13). This is especially true in our scenario, where our scores consist of closed and concise questions developed for application by doctors.

Also, there were no changes in the weights assigned to each criterion, as they were considered adequate by our endourologists. While technically this is not a fault and is supported by translation guidelines, different cultural and regional perceptions might alter these weights, which warrants further investigation. Furthermore, the lack of external validation of these scores, both in the original articles and in our study, is another limitation. However, our translation of these scores to Brazilian Portuguese is an essential first step towards future validation studies, which are currently ongoing in our institution.

There are many other aspects of these tools that are a source of discussion (16). First, time on the waiting list is not a criterion included in the scores, which may delay or even make it impossible to treat low priority patients. Many authors believe that this might constitute denial of care, and that a window to treat diseases before complications occur is being lost. For this reason, time can be used as an adjunct factor within the scores, with its weight defined according to the needs of each service (17). Also important is the lack of many criteria that might influence, even if slightly, the evaluation of patient priority such as comorbidities, socioeconomic status and age (18-20). While incorporating too many questions can make them difficult to use, it is possible that relevant criteria are being missed.

The availability of validated prioritization scores in Brazilian Portuguese enables researchers to address these unanswered questions and to investigate the efficacy of these scores in accurately assessing patients’ urgency for surgery. It also allows for the exploration of additional variables or criteria that may be specific to the Brazilian population, further refining the scoring system. It is important to note that, after the conclusion of this study, the endourology division of our institution began using the translated version of the SCQ score as shown in Figure-1 in daily practice. We opted to include the specification of "moderate or severe" upper tract dilation into the question statement to better clarify it, as is done in the other items. The statement of item 2 as we use today is shown in the supplementary material.

In conclusion, our study successfully translated and culturally adapted two previously published prioritization scores for urinary stones into Brazilian Portuguese. Despite the limitations, this work contributes to the field of surgery waiting line management in Brazil and lays the foundation for future validation studies. The availability of these scores in the local language will support evidence-based decision-making, promote equity, and improve patient outcomes in the management of surgical waiting lists.

## CONCLUSIONS

Our research successfully adapted two patient prioritization tools, the Western Canada Waiting List and the SCQ-score, into Brazilian Portuguese. These scores are user-friendly and can be completed quickly, making them practical for clinical settings. This represents an important initial advancement in the field, allowing further studies to assess the implications and benefits of utilizing these criteria in surgical waiting lists, potentially aiding thousands of Brazilian citizens.

## SUPPLEMENTARY MATERIAL

### Fictitious cases used in this study

**Table:**

**A**	Female, 77 years old Asymptomatic patient, with the finding of a 6cm staghorn stone in the left kidney. No pain. No emergency room visits. Performs household tasks and helps care for her grandchildren. CT scan shows kidney with good parenchyma (18mm) and slight dilation. ASA III, Creatinine 0.9 (creatinine clearance 66). Negative urine culture.
**B**	Female, 40 years old Cystitis (2 to 3 per year) and occasional bilateral lower back pain for a year, uses dipyrone with improvement. Reports finding of kidney stone on routine ultrasound. No emergency room visits but is very worried about her health as she has two young children. CT - left kidney with incomplete 4 cm staghorn stone, good parenchyma (18mm), and slight dilation. Right kidney normal. ASA I, Creatinine 1.0 (creatinine clearance 73). Urine culture with E. coli > 100,000 CFU and resistance to oral medications.
**C**	Male, 35 years old Right kidney pain for 3 months. Weekly visits to the ER, with worsening in the last two weeks. Using daily home analgesia with severe pain. Unable to work, cries from pain during consultation. Very worried, fears losing his job as he is the family's sole provider. CT with 2.5 cm obstructive right pelviureteric stone, with moderate hydronephrosis, delayed contrast excretion, and significant peri-pelvic inflammation. ASA I, Creatinine 1.4 (creatinine clearance 67). Negative urine culture.
**D**	Male, 35 years old With right kidney pain for 3 months. Weekly ER visits, with worsening in the last two weeks. During last ER visit 7 days ago, underwent right double J stent placement for colic. Now the pain is controlled, only feels mild discomfort when voiding and hematuria when playing soccer. Using sporadic home analgesia. Has been able to work. Worried as he has a double J and was told it could calcify soon. CT with 3 cm right pelviureteric stone, mild hydronephrosis, and well-placed double J. ASA I, Creatinine 1.4 (creatinine clearance 67). Negative urine culture.
**E**	Male, 20 years old History of cystine kidney stones since childhood, previously underwent right nephrectomy for renal exclusion due to pyonephrosis, now with intermittent and moderate left lower back pain for 3 months. Takes analgesics with good control but misses college classes 1 to 2 times a week due to pain. The family was very concerned about the possibility of him losing this kidney and becoming dialytic. CT with single left kidney, multiple calyx stones up to 1 cm and a 2 cm pelviureteric stone, parenchyma with areas of retraction, moderate dilation ASA II, Creatinine 2.0 (creatinine clearance 48). Negative urine culture.
**F**	Female, 56 years old History of recurrent kidney stones for 15 years, with bilateral lower back pain for 1 year and recurrent UTIs. Monthly emergency room visits due to severe pain and infection. Previously underwent bilateral percutaneous nephrolithotomy, having residual stones. Patient depressed as she sees no solution for her case, does not leave the house anymore. CT with incomplete bilateral staghorn stone, parenchyma with areas of retraction and moderate dilation to the left. ASA III, BMI 48, AMI 12 months ago, on clopidogrel, chronic hearth failure with ejection fraction of 50%. Creatinine 2.0 (creatinine clearance 29) and Urine culture with Carbapenem-resistant Escherichia coli.
**G**	Female, 56 years old History of recurrent kidney stones for 15 years, with bilateral lower back pain for 1 year and recurrent lower UTIs. Had an episode of obstructive pyelonephritis on the left 15 days ago, underwent percutaneous nephrostomy and antibiotic therapy. Patient with mild and constant pain at the nephrostomy site, unable to work, as she is a cook. CT with incomplete bilateral staghorn stone, parenchyma with areas of retraction and well-placed left nephrostomy. ASA II, BMI 42. Creatinine 1.6 (creatinine clearance 38). Negative urine culture.
**H**	Female, 56 years old. Patient with complete left staghorn stone, underwent PCNL 1 week ago, surgery interrupted by bleeding, currently with nephrostomy. 60% of the calculus mass was treated and new surgery is needed. Has moderate pain at the nephrostomy site and is unable to work. ASA II, BMI 35. Creat 1.2 (creatinine clearance 53), hemoglobin 12, and negative urine culture.
**I**	Female, 70 years old History of recurrent UTI for 5 years. Had a severe case of pyelonephritis 9 months ago, requiring ICU. CT at the time showed an incomplete 4cm left staghorn stones, obstructive, with signs of acute pyelonephritis. Underwent left double J stent placement and antibiotic therapy, with improvement. Returns now, with mild and occasional lower back pain, uses dipyrone with improvement, and mild but daily hematuria and dysuria. Stroke 5 years ago, partial hemiplegia, moves with difficulty, requires a caregiver at home for regular activities. Family very concerned about the situation, saying the double J was forgotten in the patient and now her condition is worse, and that the hospital should be held responsible. Current CT shows left kidney with the same renal stone, but with a 5 cm double J calcified in the bladder and the kidney, near the stone. ASA III. Creatinine 0.9 (creatinine clearance 69) and urine culture with multi-sensitive Proteus and Candida.
**J**	Female, 35 years old History of recurrent UTI since childhood. History of myelomeningocele and neurogenic bladder, performs clean intermittent catheterization every 4 hours. Moves with a wheelchair, works, and has one child. No pain and no need for emergency services, but requires frequent consultations that disrupt her daily life. Concerned about the possibility of losing her kidney. CT shows right kidney with complete staghorn stone, moderate hydronephrosis, signs of parenchymal and cortical retraction of 10mm, DMSA of this kidney at 25%. Left kidney without calculi, with good parenchyma. ASA II. Creat 1.1 (creatinine clearance 67) and negative urine culture.
**K**	Male, 18 years old Right double J placed 1 month ago due to refractory renal colic from a 1cm proximal ureteral stone. History of kidney stones for 2 years, previously underwent 2 shock wave lithotripsies and 1 percutaneous nephrolithotomy. No UTIs. Previous stone analysis confirmed Cystine. Constant and severe pain due to double J, missing classes, and may fail the year in college due to pain absences. Ultrasound with mild right hydronephrosis and multiple calculi up to 2 cm. DMSA right kidney 30% ASA II, BMI 16. Creat 1.8 (creatinine clearance 41), Hb 12, negative urine culture.
**L**	Male, 46 years old Bilateral renal colic for 30 days, with recurrent hospitalizations due to pain and general condition decline. Double J passed 1 week ago due to acute post-renal renal failure. Constant moderate intensity pain by the catheter, missed work once due to discomfort. CT with bilateral ureteral calculi (7 and 9mm) and well-placed bilateral double J. Right pelviureteric stone of 3 cm. ASA I. Cr 1.1 (creatinine clearance 63). Negative urine culture.

### Translated Version of SCQ-score

**Table:**

O Score SCQ	
1 - Infecção alta no momento da inclusão ou enquanto está na lista de espera (S/N)	NÃO = 0 SIM = 3
2 - Obstrução do trato urinário superior enquanto está na lista de espera (S/N)	NÃO = 0 SIM = 3
3 - Tempo de implante de cateter para derivação urinária (Duplo J / Nefrostomia Percutânea) A: sem catéter B: 0-3 meses C: 3-6 meses D: > 6 meses	A = 0 B = 1 C = 2 D = 3
4 - Internações hospitalares relacionadas ao caso de litíase, enquanto está na lista de espera (S/N)	NÃO = 0 SIM = 2
5 - Sintomas no momento da inclusão ou enquanto está na lista de espera (S/N)	NÃO = 0 SIM = 2
6 - Localização ureteral da litíase (S/N)	NÃO = 0 SIM = 1
7 - Rim único ou sub-ótimo (FRD < 35%)(S/N)	NÃO = 0 SIM = 2
8 - Insuficiência renal crônica (RFG < 60 mL/min.)	NÃO = 0 SIM = 1
9 - Portador de nefrostomia percutânea (S/N)	NÃO = 0 SIM = 1

**Table:**

SCQ SCORE	0 - 18
Infecção na inclusão ou enquanto está na lista de espera: sepse, pielonefrite, pionefrose (ITUs baixas são excluídas) Obstrução do TUS: dilatação moderada ou severa de, ao menos, um cálice renal sem derivação urinária ou apesar de ter derivação. Todos os exames de imagem disponíveis devem ser avaliados. Tempo de permanência de derivação urinária (duplo-J ou nefrostomia percutânea): desde sua primeira colocação, em meses. Novas internações ao hospital enquanto está na lista de espera, relacionado ao caso do cálculo Sintomas na inclusão ou enquanto está na lista de espera, relaciona ao cálculo (sintomas da derivação incluídos): hematúria, dor lombar, ITUs baixas recorrentes, disúria. Inclui pacientes precisando de afastamento ou dependentes de cuidado devido aos cálculos. Cálculo localizado no ureter Cálculo em rim único, tanto anatômico ou funcional (FRD < 15%) ou em rim sub-ótimo (FRD < 35%, se não houver DMSA disponível, responder NÃO) Doença renal crônica (DRC): RFG menor que 60 mL/min. por 3 meses ou mais. RFG calculado usando a equação CKD-EPI, disponível em https://senefro.org/modules.php?name=calcfg NPC: paciente portando nefrostomia percutânea S/N - sim/não; DJ - duplo J; FRD - função renal diferencial; TUS - trato urinário superior; ITUs - infecções do trato urinário; CKD-EPI - Chronic Kidney Disease Epidemiology Collaboration	

### Translated version of WCWL criteria

**Table:**

CRITÉRIOS WCWL		PESO
**1. Frequência habitual de episódios de dor / sofrimento**		
	Nenhum	0
	Ocasional	3
	Frequente	6
	Constante	9
**2. O quão ruim é a dor quando ela está pior?**		
	Sem dor	0
	Leve	3
	Moderada	7
	Severa	11
**3. Intensidade habitual de outras formas de sofrimento**		
	Nenhuma	0
	Leve	4
	Moderada	8
	Severa	12
**4. Grau de comprometimento das atividades habituais decorrentes da condição cirúrgica**		
	Sem comprometimento / Comprometimento leve	0
	Capaz, mas com dificuldade e/ou com comprometimento moderado	5
	Capaz, mas com muita dificuldade e com comprometimento importante	10
	Totalmente dependente (incapaz de realizar qualquer atividade habitual)	15
**5. História recente de complicações maiores ou alterações significantes em exame físico ou em resultados de exames**		
	Não	0
	Sim	8

### Statement changes in item 2 of SCQ-score

**Table:**

**Previous**	Obstrução do trato urinário superior enquanto está na lista de espera (S/N)
**Actual**	Obstrução moderada ou severa do trato urinário superior enquanto está na lista de espera (S/N)
